# Cognitive-behavioral intervention via interactive multimedia online video game for active aging: study protocol for a randomized controlled trial

**DOI:** 10.1186/s13063-019-3859-5

**Published:** 2019-12-09

**Authors:** Fernando L. Vázquez, Ángela J. Torres, Patricia Otero, Vanessa Blanco, Lara López, Antonio García-Casal, Manuel Arrojo

**Affiliations:** 1 Department of Clinical Psychology and Psychobiology, University of Santiago de Compostela, Santiago de Compostela, Spain; 2 Department of Psychiatry, Radiology, Public Health, Nursing and Medicine, University of Santiago de Compostela, Santiago de Compostela, Spain; 3 Department of Psychology, University of A Coruña, A Coruña, Spain; 4 Department of Evolutive and Educational Psychology, University of Santiago de Compostela, Santiago de Compostela, Spain; 5 Department of Psychiatry, Instituto de Investigación Sanitaria (IDIS), Complejo Hospitalario Universitario de Santiago de Compostela, SERGAS, Santiago de Compostela, Spain

**Keywords:** Older adults, Video game, Active ageing, Health promotion, Study protocol

## Abstract

**Background:**

Due to the progressive aging of the population, programs to promote active aging have been recommended. However, older adults have difficulty accessing them. Interventions administered through online video games may increase their accessibility, and complementing these with a smartphone app will likely increase adherence and allow for ongoing professional monitoring. The objective of this study is to evaluate the efficacy of a cognitive-behavioral intervention for active aging administered through an online interactive multimedia video game that includes a smartphone app companion. The secondary objectives are to analyze the moderators and mediators of the change in the outcome variables and to evaluate the adherence to the intervention.

**Methods/design:**

A randomized controlled clinical trial will be conducted. Adults 45 years and older will be randomly assigned to a cognitive-behavioral intervention administered through an online multimedia video game that includes a smartphone app companion or to a control group that will receive online information on active aging (274 participants per group). The intervention will be administered in eight weekly 45-min modules. An investigator-blinded evaluation will be conducted using online self-administered tests at baseline, post-intervention, and 6- and 12-month follow-ups. The primary outcome will be mental health status as evaluated using the 36-item Short-Form Health Survey (SF-36) at post-intervention. Secondary outcomes will be emotional well-being, depressive symptoms, reinforcement, negative thoughts, self-reported memory, cognitive task performance, sleep hygiene behaviors, physical activity, eating habits, body mass index, social support, dropout, treatment adherence, and satisfaction with the intervention.

**Discussion:**

If the results are favorable, this study would involve the development of the first evidence-based active aging promotion intervention based on a video game that includes a smartphone app companion, providing evidence on its efficacy, accessibility, and clinical utility.

**Trial registration:**

ClinicalTrials.gov, NCT03643237. Registered 27 August 2018.

## Background

In Europe, the average age of the general population is increasing, with 19.1% over the age of 65 and 39.2% over the age of 50 [1]. Among the most common mental disorders in these age groups are depression and dementia [2], which are associated with high social and health costs [3, 4]. Moreover, these costs are expected to continue to increase, given that the percentage of people over the age of 65 in Europe will increase to 28.7% by 2080 (an increase of approximately 10 percentage points) [5]. As a result, the World Health Organization (WHO) [2] has emphasized the importance of programs promoting active aging with the goal of increasing the number of healthy and independent elderly people. In addition, the WHO recommends prescribing healthy lifestyles, such as physical activity and a balanced diet, to prevent diseases and preserve physical and mental faculties [6].

However, the lack of social and health care services for older adults, especially in rural areas [7], which tend to have more elderly people [8], may limit access to health preventive programs targeting key areas such as depression, cognitive decline, and unhealthy eating habits. One way to overcome these barriers and offer a program of active aging in an attractive way is through online video games. Video games are inexpensive, fun, and can be accessed from home at convenient times [9–11]. Furthermore, middle-aged and older adults are now more digitally connected than ever. Eighty-five percent of people ranging in age from 45 to 64 years and 67.8% of people aged 65 years and older have a computer with an Internet connection [12]. In addition, 80.3% of people ranging in age from 45 to 64 years, 59.0% of 65–69 year olds, and 49.0% of 70–74 year olds have their own smartphone [13]; and this is just the beginning, since the number of older people with technological skills will even increase in the future. There is evidence supporting the effectiveness of video games in the general population for mental health [14], health promotion [9, 15], and the improvement of health-related outcomes [16]. Video game-based psychological interventions have been shown to be effective for the treatment of specific phobias [17] and depression [18] in young and middle age adults. However, the effectiveness of video games for active aging in adults from middle to late adulthood has received very little attention in the literature.

A meta-analysis comprising 21 randomized controlled trials about video game-based interventions aimed at healthy adults older than 44 found that video game-based interventions produced positive effects on objectively measured physical health, negative affect, and social health, with small to small-to-medium effect sizes (*d* = 0.34, *d* = 0.26, and *d* = 0.40, respectively) [19]. However, these interventions have not been built on current knowledge of psychological science nor are they based on theoretical models. In addition, none of the interventions were based on a manualized treatment and few were based on a protocol, which limits the generalizability of the interventions. There was a wide use of non-standardized measures, and follow-ups were scarce.

In addition, there are few studies on serious video games or programs with gaming features aimed to promote behavior change and/or health [20], and they were only focused on cognitive training. There was only one video game for the prevention of depression in older people [21] and the games directed at physical condition have focused on balance and action [22]. None of the games have targeted developing a habit of physical activity and they have ignored other fundamental aspects related to health such as diet. Furthermore, none of the aforementioned studies integrated the components of mood, cognitive stimulation, and healthy life habits in the same intervention, despite the fact that a review of modifiable risk factors for dementia estimated that 51% of population-attributable risk was associated, among other factors, with depression, cognitive inactivity, physical inactivity, and midlife obesity; and these midlife risk factors could be tackled early to prevent or delay the onset of dementia [23]. In addition, none of the studies used a story or narrative thread in the video game, despite the fact that immersion in the history of the game can facilitate change of behaviors related to health [24] and that video games with storylines might be preferred by the elderly [25].

Finally, these studies had dropout rates of up to 25% [19] and reported insufficient playing time, a potential reason for lack of efficacy of video game-based interventions [15]. Thus, an additional device (such as a smartphone app) that complements the video game by sending reminders in real time and that allows for a follow-up by a clinical professional could help increase adherence to the intervention and the generalization of the video game to real life (i.e., using the skills learned in the video game in daily real life situations beyond the gaming sessions).

### Aims and hypothesis

The main objective of the randomized controlled trial is to evaluate the efficacy of a cognitive-behavioral intervention to promote active aging in a holistic manner (incorporating mental health status, emotional well-being, depressive symptoms, self-reportd memory, healthy habits, and social support). The intervention will be administered through a serious online interactive multimedia video game with a complementary smartphone app. The intervention group will be compared to a control group that will have access to online information. The secondary objectives are to analyze the moderators and mediators of the change in the outcome variables and to evaluate the adherence with the intervention. As a central hypothesis, the intervention is expected to significantly improve mental health status compared to the control group. In addittion, intervention is expected to improve emotional well-being, depressive symptoms, self-reported memory, sleep hygiene behaviors, physical activity, eating habits, body mass index, and social support. As secondary hypotheses, it is expected that (a) the baseline values on sociodemographic variables, emotional well-being, depressive symptoms, self-reported memory, sleep hygiene behaviors, physical activity, eating habits, body mass index, and social support will moderate the effects of the intervention, and that reinforcement, negative automatic thoughts and cognitive task performance will mediate the effects of the intervention, (b) the experimental group will show a lower percentage of dropouts and higher satisfaction than the control group, and (c) cognitive task performance and treatment adherence will be higher than 50%.

## Methods/design

### Design

A randomized controlled clinical trial will be conducted. The study protocol is in accordance with the Standard Protocol Items: Recommendations for Interventional Trials (SPIRIT) guidelines (see checklist in Additional file 1). Participants will be randomly assigned to: (a) an experimental group (EG) that will receive a cognitive-behavioral intervention administered through a serious online interactive multimedia video game with a complementary smartphone app for active aging (CBI-V) or (b) a control group (CG) that will receive online information on active aging.

**Fig. 1:**
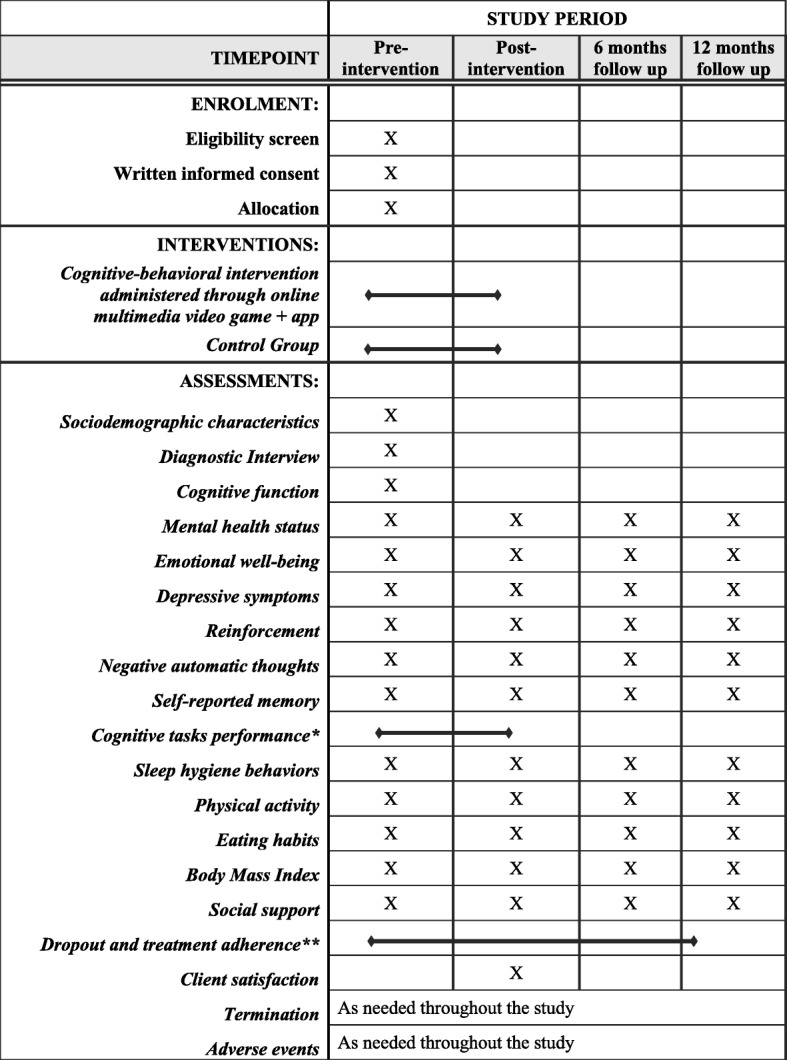
SPIRIT figure. Phases of the randomized controlled trial

The stages of the study are shown in Fig. 1. A combination of interviewer-based and self-report measures will be administered at pre-intervention, post-intervention (assessment will be performed at the end of the intervention, i.e., from 8 to 16 weeks after the beginning of the intervention), 6- and 12-months follow-up.

### Participants

#### Recruitment

The research team of the University of Santiago de Compostela and the Ramón Domínguez Foundation will recruit the study participants from the population of people aged 45 years and over through clinics and health centers in the Autonomous Community of Galicia (Spain). Galicia is a region in the northwest of Spain with an area of 29,434 km^2^ and a population of 2,730,337 inhabitants (of whom two-thirds live in councils of less than 50,000 inhabitants) and it has the second oldest population in Spain [26]. Interested persons will be contacted and they will be given a brief description of the study. If they meet the inclusion criteria, they will be invited to participate in a complete evaluation that will include an interview by independent evaluators to ensure the lack of mental disorders and assess their cognitive status to adhere to the exclusion criteria. In addition, they will complete an online evaluation that will include the assessment of sociodemographic characteristics, mental health status, emotional well-being, depressive symptoms, reinforcement, negative thoughts, self-reported memory, sleep hygiene behaviors, physical activity, eating habits, body mass index, and social support by means of self-administered instruments. The potential participants will be asked to give their written informed consent before being randomly assigned to a study group.

#### Eligibility criteria

To participate in the study, participants will be required to (a) live in the Autonomous Community of Galicia, (b) be 45 years or older, (c) have the appropriate devices to play the game (computer, Smartphone and Internet connection), (d) give written consent to participate in the study, and (e) commit to complete the program and subsequent evaluations. We will exclude those participants who (a) have serious medical or mental disorders (e.g., depression, schizophrenia, bipolar disorder, major neurocognitive disorder, dissociative disorders, substance dependence or abuse), (b) have sensory, physical, or mental problems that make it impossible to complete the study (e.g., mild neurocognitive disorder, severe visual or auditory impairment), (c) have received psychological or psychopharmacological treatment in the last 2 months, and (d) are participating in another study.

#### Randomization

Stratified randomization by age groups will occur after screening and baseline assessments. Specifically, the age groups are 45–64 and ≥ 65 years old, consistent with Levinsons’ life cycle theory [27]. An independent researcher (allocation concealment) will make random allocation cards using computer-generated random numbers. The original random allocation sequences will be kept by the independent researcher in an inaccessible third place and the researcher will work with a copy. The randomization sequence will be communicated to the researchers by means of sealed numbered envelopes, one for each participant, with instructions to use them in numerical order. However, participants cannot be blinded to their treatment allocation due to the nature of the interventions.

#### Sample size calculation

Assuming a two-tailed test, α of 0.05, a power (1 − β) of 0.80, and a proportion of subjects in each group of 0.5, we estimated that a sample size of 253 participants per group will be required to detect a small effect size of 0.25. This effect size is the minimum needed to reach meaningful clinical change and is consistent with the smallest effect size found in a previous meta-analysis of video game-based interventions for active aging [19]. Specifically, an effect size of 0.26 for the mood outcome in active aging video game-based interventions was the smallest effect size identified [19]. To safeguard against an estimated 8% attrition rate [19], a minimum of 274 in each group must be recruited, which means 548 participants in total (550 approximately).

### Ethics

The study procedures follow the principles of the Declaration of Helsinki and have been approved by the Bioethics Committee of the University of Santiago de Compostela (Spain). Any significant amendment to the protocol will have to be approved by the Committee prior to its implementation. Participation is voluntary, without incentives of any kind, and all participants will give their written informed consent. If a mental disorder is detected during any evaluation, the participant will be contacted to explain the finding and a referral to appropriate care will be provided. Consistent with the inclusion/exclusion criteria, the participant will be excluded from the study. After the completion of the study, any necessary or requested post-trial ancillary care will be attended and managed by the steering committee.

A comprehensive attempt will be made to measure adverse events. Participants will be asked to report adverse events by phone as soon as possible after they have occurred. Any adverse effects (muscle pain, visual fatigue, etc.) during the study will be recorded on a standardized adverse event form by the staff member and reported to the study coordinator. Subsequently, adverse events will be reported to the steering committee, who will take the necessary steps and provide details in the final report of the study.

### Interventions

The EG intervention will be administered through a serious online interactive multimedia video game and the CG materials through online readings. The same software and the same content will be administered to all the participants assigned to the same condition, which guarantees homogeneity in the application and increases the internal validity of the study. All participants will be trained in a 30-min session and will receive a personalized instruction booklet with illustrations about how to log on to the online platform and how to report technical problems. The EG will be shown how to play the game and the CG will be shown how to access the active aging information within the platform.

#### Cognitive-behavioral intervention administered through online interactive multimedia video game with smartphone-based app companion

The EG intervention will be administered through a serious online interactive multimedia video game on a computer and a complementary smartphone-based app. The app allows the extension of the user’s training, monitoring their progress and sending reminders in real time, as well as sending feedback to the clinicians about their progress. The contents have been designed by a team of expert psychologists from the University of Santiago de Compostela (Spain) and the video game has been developed by the informatic and telecommunications solutions companies Imatia Innovation, Sivsa Informatic Solutions, and Gradiant (Spain).

The intervention will be delivered based on a standardized treatment manual. The intervention consists of eight modules of approximately 45 min each. The modules will be administered at a rate of one per week with between-session homework to practice the trained skills in real life. The modules can also be administered one every two weeks if the participant needs more time to complete the homework. Thus, the intervention can last between 8 and 16 weeks. The video game is a graphic adventure, with a narrative thread or story that involves the player’s experience and depicts a story, based on the French Way of St. James (i.e., the pilgrimage road from Roncesvalles –France– to the cathedral of Santiago de Compostela –Spain–).

The intervention consists of three components: prevention of depression, cognitive stimulation, and promotion of healthy lifestyle habits (sleep, physical activity, and diet). The prevention of depression component was adapted from an indicated-prevention intervention for depression based on the multi-factorial etiopathogenic model of depression by Lewinsohn et al. [28]. This intervention has been shown to be effective in the prevention of new episodes of major depression and in the reduction of depressive symptoms in the short and long term in both face-to-face format [29, 30] and by telephone multiconference [31]. The cognitive stimulation component of the intervention is based on the notion of “brain reserve” [32] and the concept of “cerebral neuroplasticity” [33] and is based on the model of cognitive stimulation developed by Spector et al., including mental stimulation, seeking new ideas, thoughts, and associations, and using hints and implicit learning [34, 35]. In addition, possible causes of memory deficits associated with aging are considered in the intervention: decreases in processing resources, lack of semantic coding, metamemory problems, and difficulties in the deliberate retrieval of information [36]. Finally, the promotion of healthy lifestyle habits component is based on learning theory and social cognitive theory [37], through which many human behaviors are learned via observation by way of attention, retention, production, and motivation. The change in behavior is a function of the state of skill development and confidence (self-efficacy) in performing the new behavior, with modeling, self-control strategies, goal setting, and feedback fundamental techniques for learning [38].

As shown in Table 1, throughout the intervention, the main character (Jacob) walks along the French Way of St. James meeting different characters that teach him different techniques to encourage changes that promote well-being and increase quality of life. In the first module, the concept of depression and the need for active coping for depressive symptoms are explained, and diaphragmatic breathing is also introduced. The second module focuses on increasing pleasant activities and self-reinforcement. From the third to the fifth module, participants are introduced to healthy sleep habits, physical activity, and healthy eating and given guidelines for implementing healthy habits. In the sixth and seventh modules, participants are trained in the identification of cognitive distortions and cognitive restructuring. The eighth module focuses on self-esteem and relapse prevention. Cognitive stimulation, social skills, self-efficacy, and a sense of control are transversal content to all modules.

**Table 1:** Content of the video game-based cognitive-behavioral intervention for active aging

Module/component	Narrative	Techniques and strategies	App companion support
1 Mental health, cognitive stimulation	*From Roncesvalles to Pamplona* Presentation of the main character (Jacob) and start of the trip. Jacob is at a stage in which he feels older, tired, sad, does not sleep or eat properly, does not perform physical exercise. He starts the French Way of St. James to find the peace he needs. An old man invites him to take a book to guide him through the stages. The book will show him the way and will give him knowledge to reach his personal goal. To begin, it explains the relationship between our behaviors, thoughts, and emotions. He meets Txomin, who teaches him to monitor his emotions and a group of pilgrims who teach him a relaxation technique. He must pass through challenges (microgames of cognitive stimulation) to continue the path. He meets the first illustrious pilgrims (e.g., Jean de Brienne), historical figures (e.g., King Alfonso II, Isabel the Catholic), and visits places of interest along the way: Albergue de Roncesvalles, Puente de Zubiri, Albergue Casa Paderborn.	-Presentation of the protagonist and The Way. -Active aging: importance of emotional, physical, and cognitive well-being for healthy active aging. -Mental health: explanation of mood problems and the need for active coping for optimal mental health; mood state diary. -Healthy habits: training in relaxation (diaphragmatic breathing) through modeling. -Cognitive stimulation: mnemonic strategies, verbal and visual memory tasks. -Social skills, assertive communication.	-Review of what has been learned. -Map of the path: shows progress and places and significant learning at this stage of the trip. -Homework (with reminder notifications). - Mood tracking (self-report). - Practice relaxation (diaphragmatic breathing technique). -Trophies: can be obtained through adherence and weekly compliance with tasks.
2 Mental Health, cognitive stimulation	*From Pamplona to Estella* He meets the legend of the Source Reniega. Belfegor appears (minion of Lucifer) and tries to snatch the book. The Apostle James comes to his defense. Jacob is attacked and falls unconscious and in dreams he performs a challenge in order to continue on his way. He gets the indication to search and find nine groups of emotional vitamins in the road symbols (scallops); to get hold of them, he must pass several challenges (microgames of cognitive stimulation). Another pilgrim teaches him the value of self-reinforcement and how to put it into practice. He visits places of interest along the way: Source Reniega, Alto del perdón, Brigde and Square de Puente la Reina, Church and hospital of pilgrims of Estella.	- Positive or corrective feedback of weekly homework compliance. -Review of contents of Module 1. Long-term memory exercises. - Mental health: effect of pleasant activities on mood, guidelines for identifying personal pleasant activities; self-reinforcement. - Cognitive stimulation: mnemonic strategies, verbal fluency tasks, visual tracking, cancellation, ideomotor praxia, verbal and visuospatial short-term and working memory. -Social skills, assertive communication.	Previous and: - List of personal pleasant activities.
3 Mental Health, cognitive stimulation, sleep habits	*From Estella to Nájera* Jacob does not sleep well. He is tired, but various characters like Hirobumi and other pilgrims give him guidelines to improve his sleep habits. He is reunited with Txomin, who teaches him to plan pleasant activities and make contracts with himself. By doing this he declares, unknowingly, the war on Disnergia (fantastic character), who wants his book. In order to fight against her, he must get the cross of Tau, and to achieve it, he must solve microgames of cognitive stimulation. A battle with Disnergia takes place, and he expels her by performing a challenge He visits places of interest along the way: Pamplona Park, Logroño.	-Positive or corrective feedback about the completion of the weekly homework. - Review of contents of Module 2. Long-term memory exercises. - Mental health: scheduling pleasant activities. -Healthy sleep habits: discovering sleep hygiene guidelines. -Cognitive stimulation: mnemonic strategies, verbal reasoning, working memory, executive functions (planning) and spatial orientation. -Social skills, assertive communication.	Previous and: - Perform the weekly pleasant activities schedule.
4 Mental Health, cognitive stimulation, physical activity	*From Nájera to Burgos* His mood is improving. Jacob and Guillen (another pilgrim who accompanies him throughout this stage of the journey and the next) are victims of the theft of the book by Disnergia and Morbilius (fantastic characters). Templar monks help them chase them away and give them physical activity guidelines to stay in good physical condition. He must solve puzzles to unlock information. The confrontation with Disnergia takes place and he defeats her doing a challenge, but not Morbilius, who escapes. He recovers his book. He visits places of interest along the way: Botanical park of La Rioja, Hayedo de Urrez, San Juan de Ortega Church.	-Positive or corrective feedback about the completion of the weekly homework. - Review of contents of Module 3. Long-term memory exercises. - Mental health: scheduling pleasant activities. -Physical activity: essential information and intrinsic motivation (choosing personal value statements to improve this area); setting of a final goal and graduated subgoals; planned action (what, with whom, when, where, and how). -Cognitive stimulation: verbal work memory, executive functions (planning), ideational praxia. - Social skills, assertive communication.	Previous and: - Perform the plan to achieve the weekly goal of physical activity; self-monitoring and self-reinforcement.
5 Mental health, cognitive stimulation, physical activity, healthy eating habits	*From Burgos to Sahagún* The monks tell him that a wise man knows how to defeat Morbilius. The book, after conducting challenges (micropuzzles of cognitive stimulation) gives clues about the wise man and healthy eating patterns. They go to a restaurant to eat, where they meet the wise man and a woman (Venustea), but Morbilius kidnaps her and takes the book away, and they must help the wise cook by taking memory challenges related to information about food and memory. They get objects that will help them fight against Morbilius. They fight with Morbilius, save Venustea and recover the book. Guillen decides to stay with Venustea and Jacob continues on his way. He visits places of interest along the way: San Tirso de Sahagún Church.	-Positive or corrective feedback about the completion of the weekly homework. - Review of contents of Module 4. Long-term memory exercises. -Healthy eating habits: essential information and intrinsic motivation (choosing personal value statements to improve this area); eating pyramid (food groups and frequency intake); meal planning (portions and cooking). - Cognitive stimulation: mnemonic strategies, visual and work memory, ideomotor praxis, executive functions (planning). - Social skills, assertive communication.	Previous and: -Daily record of food intake. - Healthy eating plan: changes according to three priority levels.
6 Mental health, cognitive stimulation, physical activity, healthy eating habits	*From Sahagún to Rabanal* The book provides an explanation of the thoughts and their influence on mood. He meets the architect Antón Gaudín (historical character), whom he helps rid of cognitive distortions by overcoming challenges. The book teaches him to identify and record their own negative thoughts. He visits places of interest along the way: Pantheon Museum of the Royal Collegiate of San Isidoro de León, Episcopal Palace of Astorga, Rabanal Hostel.	-Positive or corrective feedback about the completion of the weekly homework. - Review of contents of Module 5. Long-term memory exercises. - Mental health: connection situation-thought-emotion; the effect of negative thoughts on mood; main cognitive distortions (e.g., overgeneralization); self-registration of negative thoughts. - Cognitive stimulation: attention and concentration, language, verbal and visual memory, ideomotor praxia. - Social skills, assertive communication.	Previous and: - Negative thought detection.
7 Mental health, cognitive stimulation, physical activity, healthy eating habits	*From Rabanal to Triacastela* He finds a muleteer in his trip (a carrier of merchandise in a cart drawn by horses) when he is attacked by the cognitive distortions (minions of Lucifer). The muleteer, and later a Templar, teach him to substitute his negative and irrational thoughts for more rational and positive ones. Finally, Jacob helps the muleteer when he is attacked. He visits places of interest along the way: El Bierzo, Ponferrada, O Cebreiro, Triacastela.	-Positive or corrective feedback about the completion of the weekly homework. - Review of contents of Module 6. Long-term memory exercises. - Mental health: cognitive restructuring strategies (direct approach, the worst that could happen, opinion survey, how does this thought help?). - Cognitive stimulation: attention, working memory, long-term memory, ideomotor praxia, cognitive functions (planning). - Social skills, assertive communication.	Previous and: - Use of cognitive restructuring strategies record.
8 Mental health, cognitive stimulation, physical activity, healthy eating habits	*From Triacastela to Santiago* He meets again with the muleteer, who gives him a valuable object: the cognitero, similar to the botafumeiro that serves to keep the treasures of our mind. He is reunited with Hirobumi, who gives him the seed of the tree of self-esteem and teaches him how to strengthen it so that it grows healthy and strong. Txomin teaches him the priming technique to inject positive thoughts. Before reaching the goal, he fights the boss (Lucifer); all the characters whom Jacob met along the way appear, contributing their teachings and thus helping to defeat Lucifer. Jacob makes an offering to Santiago, who gives him the Compostela (a document given to all pilgrims who complete the pilgrimage) in recognition for having completed the path and acquire healthy lifestyle habits. Jacob prepares himself for a new life with more illusion; he knows himself better and has the strategies to live happier. He visits places of interest along the way: Monte del Gozo, Cathedral of Santiago de Compostela.	-Positive or corrective feedback about the completion of the weekly homework. - Review of contents of Module 7. Long-term memory exercises. - Mental health: techniques for managing thoughts (distraction, priming); self-esteem enhancement (identifying personal strengths and achievements). -Cognitive stimulation: attention, ideomotor praxis, language, working memory, planning, long-term memory. -Social skills, assertive communication. - Progress maintenance, farewell and closing.	

*Note*: Throughout the game, when participants pass each challenge they get points (positive reinforcement). The challenge gradually increases the level of difficulty

#### Online information control group

Participants in the control group will have access to an online platform that contains material with general non-specific information on active aging, structured in eight modules of approximately 45 min each at a rate of one per week. In particular, the information will cover general information related to aging, depression, cognitive deterioration, healthy lifestyle habits such as physical exercise, sleep, and balanced nutrition, and social relationships, but without guidelines for implementing them. There will be no training of specific psychological techniques and no homework assignments between sessions. Thus, this comparison group controls for contextual and non-specific factors (e.g., receiving attention, using a technological device). This results in a conservative estimate of the effect of the treatment [39].

#### Intervention adherence

All study interviewers and staff will be provided with guidelines for communication with participants to establish a good rapport and to improve adherence to the study protocol. All participants will be given the contact details of the study coordinators and be instructed to communicate any difficulties they may experience or questions that may arise during the study. To minimize the loss of participants, we will follow the strategies recommended by Grady et al. [40]; that is, making the intervention accessible and conducting non-invasive, useful, and interesting evaluations. The data collected from participants who prematurely finish the study will be registered in a Termination Form.

### Outcome measures

To evaluate if the participants meet the exclusion criteria, a diagnostic interview and an evaluation of cognitive function will be conducted. For the diagnostic interview, the Spanish version [41] of the Mini International Neuropsychiatric Interview [MINI]) [42] will be used; this is a structured diagnostic interview that explores the main mental disorders of Axis I of the DSM-IV or ICD-10. The MINI must be conducted by a clinician and has adequate validity and reliability [43]. Cognitive function will be assessed with the Spanish version [44] of the Mini-mental State Examination (MMSE) [45], which consists of 30 items, with norms adjusted for age and education, good reliability values, a sensitivity of 89.8%, and a specificity of 75.1%. The evaluation will be conducted face-to-face by trained interviewers who will not know the objectives of the study, what interventions will be administered, and randomization outcomes. The training of the evaluators will be conducted by two researchers with more than 20 years of experience in evaluation, and it will consist of 15 h of theoretical-practical seminars and role-playing about the diagnostic interview and evaluation strategies.

The participants who meet the eligibility criteria will complete the self-administered instruments through a smartphone app, and the data will be automatically downloaded into a database. Assessments will be performed pre- and post-intervention (which will be performed at the end of the intervention; that is, 8 to 16 weeks after the beginning of the intervention) and at 6- and 12-month follow-ups. Table 2 presents a synthesis of the variables, the measurement instruments, and the form of administration and Fig. 1 shows the timing for each outcome variable.

**Table 2:** Overview of measures

Instrument	Format
Screening	
Diagnostic interview: MINI	Hetero-administered (face to face)
Cognitive function: MMSE	Hetero-administered (face to face)
Participant characteristics	
Socio-demographic characteristics	Self-administered (app)
Primary outcomes	
Mental health status: SF-36	Self-administered (app)
Secondary outcomes	
Emotional well-being: GHQ-12	Self-administered (app)
Depressive symptoms: CES-D	Self-administered (app)
Reinforcement: EROS	Self-administered (app)
Negative automatic thoughts: ATQ	Self-administered (app)
Self-reported memory: MMQ	Self-administered (app)
Cognitive tasks	Registered automatically by app
Sleep hygiene behaviors: SHI	Self-administered (app)
Physical activity: BPAAT	Self-administered (app)
Eating habits: REAP-S	Self-administered (app)
Body mass index: BMI	Self-administered (app)
Social support: Duke-UNC-11	Self-administered (app)
Dropout	Registered by researcher
Treatment adherence	Registered automatically by app
Client satisfaction: CSQ-8	Self-administered (app)
Termination form	Registered by researcher
Adverse event form	Registered by researcher

*Abbreviations*: *ATQ* Automatic Thoughts Questionnaire, *BMI* body mass index, *BPAAT* Brief Physical Activity Assessment Tool, *CES-D* Center for Epidemiologic Studies Depression Scale, *CSQ-8* Client Satisfaction Questionnaire, *Duke-UNC-11* Duke-UNC Functional Social Support Questionnaire, *EROS* Environmental Reward Observation, *GHQ-12* General Health Questionnaire, *MINI* Mini International Neuropsychiatric Interview, *MMQ* Multifactorial Memory Questionnaire, *MMSE* Mini-Mental State Examination, *REAP-S* Rapid Eating and Activity Assessment for Participants-Shortened Version, *SF-36* 36-Item Short-Form Health Survey, *SHI* Sleep Hygiene Index

#### Primary outcomes

Mental health status (specifically at post-intervention) will be the primary outcome, measured with the Spanish version [46] of the 36-item Short-Form Health Survey (SF-36) [47]. This 36-item scale assesses perceived health in eight dimensions (general health, physical functioning, role-physical, bodily pain, vitality, social functioning, role-emotional, and mental health) and an item about declared health evolution, with internal consistency ranging from 0.71 to 0.94 [47] grouped in two or components (physical health and mental health) with an internal consistency of 0.94 and 0.89, respectively [48]. The eight dimensions and the two components could be used as secondary outcomes or in exploratory analyses.

#### Secondary outcomes

Socio-demographic variables will be collected through a self-administered questionnaire that will include sex, age, marital status, social class, income, level of studies, main activity, and area of residence. Marital status, social class, income, level of education, main activity, and area of residence will be recoded as binary variables. Specifically, marital status will be categorized as single, with a partner; social class as low/middle-low/middle, middle-high/high; income level as ≤ 1999€, ≥ 2000€; level of education as primary/secondary, university; main activity as domestic work/unemployed, employed; and area of residence as rural, urban. Emotional well-being will be measured using the Spanish version [49] of the General Health Questionnaire (GHQ-12) [50], consisting of 12 items, which is intended to screen for general (non-psychotic) psychiatric morbidity, whose internal consistency is 0.86 for people under 65 years and 0.90 for people aged 65 years and over. Depressive symptoms will be assessed with the Spanish version [51] of the Center for Epidemiologic Studies Depression Scale (CES-D) [52], consisting of 20 items and with a high internal consistency of 0.89. Reinforcement of the environment will be assessed with the Spanish version [53] of the self-reported Environmental Reward Observation Scale (EROS) [54]. It consists of ten items in which the participant evaluates the degree of positive reinforcement received contingently from their environment. The internal consistency of the Spanish version is 0.86. Negative automatic thoughts will be evaluated through the Spanish version [55] of the Automatic Thoughts Questionnaire (ATQ) [56]. This is a 30-item self-report questionnaire with an internal consistency of .96. Self-reported memory will be measured with the Multifactorial Memory Questionnaire (MMQ) [57], consisting of 57 items assessing three dimensions: contentment, ability and strategy. The MMQ has high internal consistency for its three subscales (0.95, 0.93 and 0.83 respectively). The number of cognitive tasks completed in the video game will also be evaluated. Participants’ sleep hygiene behaviors will be assessed with the Sleep Hygiene Index (SHI) [58], a 13-item instrument with internal consistency of 0.66. Participants’ physical activity will be assessed with the Spanish version [59] of the Brief Physical Activity Assessment Tool (BPAAT) [60], a two-item test that classifies participants into sufficiently active or insufficiently active, with good test–retest reliability (k = 0.70) and convergent validity with the International Physical Activity Questionnaire (k = 0.64) [61].

Eating habits will be assessed with the Rapid Eating and Activity Assessment for Participants-Shortened Version (REAP-S) [62]. This 16-item scale has good convergent validity with the Block Semi Quantitative Food Frequency Questionnaire (*r* from − 0.384 to 0.506) [63] and is based on the Dietary Guide-lines of the US Department of Health and Human Services [64] and the Healthy People 2010 objectives (*r* from − 0.039 to 0.516) [65]. Body mass index (BMI) is a measure for indicating body fat in adults. It is defined as an individual’s weight in kilograms divided by the square of their height in meters (kg/m^2^). Body fat is classified as underweight, normal weight, pre-obesity, and obesity (I, II, and III) [66]. Social support will be assessed with the Spanish version [67] of The Duke-UNC Functional Social Support Questionnaire (Duke-UNC-11) [68], an 11-item test with high internal consistency (0.90). Dropouts and treatment adherence will be assessed. A record will be kept of the dropouts in each group throughout the study. In addition, adherence will be assessed through numbers of modules completed, time played, and between-session homework accomplishment. Adherence will be automatically registered and monitored through the online platform and the smartphone app companion.

Finally, satisfaction with the intervention will be assessed with the Spanish version [69] of the Client Satisfaction Questionnaire (CSQ-8) [70], an eight-item scale with an internal consitency of 0.80.

### Data management

The personal data and the clinical data of the participants will be coded and saved separately. The files of the participants will be stored in numerical order for a period of 5 years after the end of the study. All data will be entered into a database where the personal data of the individuals will not be recorded. Range checks and consistency checks against data already stored in the database will be made. All evaluation and hardware related to the study data will be kept in closed cabinets. Access to the study data will be restricted to researchers through a password system. A backup copy of the original database (primary database) will be made every 15 days. All reports and publications derived from the study will be prepared so that no participant can be identified.

### Statistical analyses

We will use the SPSS statistical package for Windows (version 22.0) for the analysis of the data. All analyses will be carried out in accordance with a modified intention to treat principle. If a mental disorder is detected at post-intervention or any follow-up, the participant will be excluded from the study retrospectively [71]. If participants withdraw from the study or data are missing for another reason (e.g., incomplete questionnaires, exclusion after baseline participation due to the onset of a mental disorder), the lost values ​​will be imputed using multiple imputation [72]. Multiple imputation will be based on predictors of the outcome, including auxiliary variables (i.e., sex, age, level of studies, income, main activity, emotional well-being, depressive symptoms, reinforcement, negative automatic thoughts, self-reported memory, sleep hygiene behaviors, physical activity, eating habits, body mass index, and social support) using chained equations and the number of imputations following recommendations of Buuren [73] and White et al. [74]: using the Bodner’s approximation related to Fraction of missing information (FMI) they suggest the rule of thumb that m should be similar to the fraction of missing cases so m is selected based on FMI/m ≈ 0.01. For FMI = 0.05, 0.1, 0.2, 0.3, 0.5 the required m ≥ 3, 6, 12, 24, 59, respectively. For evaluating the severity of missing data problem (i.e., how much variability in parameter estimation is introduced by imputation) the relative increase in variance due to non-response, *ratio r* [75], and the fraction of missing information, FMI or *γ* parameter [73], will be estimated. Following recommendations of Li et al. [76], values for the FMI parameter of up to 0.2 will be interpreted as “modest”, 0.3 as “moderately large”, and 0.5 as “high”. Futhermore, Graham’s recommendations [77] for reducing biasing effects due to MNAR missing data will be followed. Therefore, auxiliary variables will be included, longitudinal missing data diagnostics will be conducted (i.e., examining the patterns of change over time), intent to drop out will be measured, and follow-up data from those missing will be collected, if possible. In addition, complete case analyses will be analyzed and will be presented in a sensitivity analysis.

To analyze the effect of the intervention on the outcome variables, linear mixed models (LMM) will be used [78, 79]. Condition x Time interaction will be analyzed through conditional F-test with degrees of freedom correction developed by Kenward and Roger [80]. When adjusting for multiple comparison the Benjamini-Yekutieli false discovery rate-controlling method [81] will be used for controlling type I error. After fitting the LMM model, residual diagnostic and influence diagnostics of unusual values will be evaluated. The assumptions should be checked for within-group error as well as for random effects following the recommendations of Pinheiro and Bates [79]. If residual checking is not satisfactory in terms of presence of heterocedasticity, different variance structures should be checked. Problems related to non-normal residuals should be avoided by changing the distribution family or using Box-Cox transformations. If problems arise from the presence of dependence in correlation, the inclusion of an adequate correlation structure will be analyzed. The effect size will be calculated using the Cohen’s *d*, interpreting values *d* = 0.20–0.49 as small, *d* = 0.50–0.79 as medium, and *d* ≥ 0.8 as large [82].

The impact of potential moderators which may influence the change in pre-/post-intervention and pre-intervention/12-month follow-up mental health will be explored with linear regression analyses. For evaluating potential moderator effects, the linear model regression proposed by Baron and Kenny [83] was applied: O = α + β_1_T + β_2_M + β_3_TM, where O represents the outcome, T represents reception of treatment, M represents the potencial moderator, and TM represents the interaction between treatment and potential moderator. Putative moderators will be baseline values of sex, age, level of education, emotional well-being, depressive symptoms, self-reported memory, sleep hygiene behaviors, physical activity, eating habits, body mass index, and social support. Variables will be centered following Kraemer and Blasey [84] recommendations.

To analyze potential mediating variables, we will use differences in pre-/post-intervention and pre-intervention/12-month follow-up mental health as the dependent variable (Y), the experimental condition received as the predictor (X), and differences in pretest–post-test levels of reinforcement and negative automatic thoughts, as well as cognitive task performance as possible mediators (M). We will fit three regression equations: Y = α + cX + ε (association between the predictor and the dependent variable); M = α + aX+ ε (association between the predictor and the mediator); Y = α + bM + c’X + ε (association between the mediator and the dependent variable controlling for the predictor). Following Hayes’s recommendations [85], the mediation effect will be estimated as c—c‘= ab, and bootstrapping will be used to determine the significance of that value. The proportion of the total effect of the predictor in the dependent variable that can be explained by the mediators will be calculated using the formula [(ab/ab + c’) × 100.

Sex and level of education will be used as binary variables, body mass index as a categorical variable (underweight, normal weight, pre-obesity, and obesity); while age, emotional well-being, depressive symptoms, reinforcement, negative automatic thoughts, self-reported memory, cognitive task performance, sleep hygiene behaviors, physical activity, eating habits, social support, adherence, and satisfaction will be used as continuous variables.

Dropouts and adherence, as well as the level of satisfaction with the intervention, will be analyzed through frequency distributions. Characteristics of patients lost to follow-up will be analyzed and compared to patients who finish the interventions to search for any attrition bias.

### Monitoring

A Data Monitoring Committee (DMC) independent of the organizers of the study formed by the Center for Industrial Technological Development will be established to monitor the correct execution of the study. This committee will be able to order independent audits once a year. The steering committee, directed by the principal investigator and consisting of representatives of Imatia, Sivsa, Gradiant, Ramón Domínguez Foundation, and the research team of the University of Santiago de Compostela, will follow the principles of good clinical practice, including quality control of the clinical protocol, the handling of the data, and the organization of the team meetings. A confidential annual report on the development of the trial will be sent to the DMC. An independent statistician will conduct interim analysis and inform the independent DMC, who will discuss the results of the analysis with the steering committee at a joint meeting. The steering committee will then decide on the continuation of the trial and report to the Bioethics Committee.

### Exit strategy

An exit strategy will be implemented in the following situations: (1) A participant decides to exit the trial prematurely. In this case, a questionnaire to find out the reasons for exit will be carried out in a (voluntary) exit phone call. Staff involved in the project will make sure the project has come to a satisfactory closure and the participant feels comfortable with the situation. (2) The trial period has come to an end. From the beginning of the study, it will be made clear to the participant that the trial period is a maximum of 16 weeks of intervention plus 12 months of follow-up. Participants will also be prepared for transition and closure.

## Discussion

In this study, the efficacy of a cognitive-behavioral intervention administered through an online interactive multimedia video game for active aging will be evaluated. Based on the results of previous studies from which the depression prevention component will be adapted [29–31], and on previous research on using video games to improve cognitive functions and promote healthy lifestyles in people of all ages [9, 20] and in older people [19], we expect to find an improvement in the mental health status, emotional and cognitive state, adoption of healthy lifestyle habits, and social support.

Previous randomized controlled trials on video game-based interventions for older adults found positive effects on emotional well-being [86, 87], cognitive functioning [88, 89], and physical health [87, 90]. Although there have been no previous interventions to reduce the incidence of depression in adults through video games based on the model of Lewinsohn et al. [28], the efficacy of this model for reducing depressive symptoms and reducing the incidence of depression has been shown in face-to-face formats and telephone multiconferencing [29–31]. Improvements in specific cognitive areas have been reported in attention, visual memory, working memory, and processing speed [91] for video game-based interventions. Similarly, previous research has found that video games for promoting healthy lifestyles have had an effect on the intention to change health behaviors [9], which are predictors of behavior change [92]. In addition, health interventions applied through video games have been shown to be as effective as other non-gamified interventions administered by computer [9].

The use of the video game format is justified for several reasons: (a) it includes characteristics such as images, sound, movement, and feedback that capture attention, the first step necessary for learning behaviors according to social cognitive theory [37]; (b) it is more attractive and rewarding than traditional face-to-face interventions [91]; (c) it increases intrinsic motivation [93], which is fundamental in the maintenance of health behaviors that entail some effort; (d) the immersion in the story allows the player to identify with the thoughts, emotions, and actions of the character of the video game, favoring commitment to intervention and changing attitudes and behaviors related to health [24]; (e) it provides the opportunity to plan behavioral changes and test them in a safe and relaxed environment [94]; (f) the immediate and concrete feedback provided by the video game reinforces the continued effort and keeps the player within a *zone of proximal development* [95], cultivating a persistent and optimistic motivational style [96]; and (g) the modeling and the repetitive nature of the video game favor learning and the acquisition of new behaviors [97].

Furthermore, the smartphone app complementary to the game is an innovation compared to previous video game-based interventions and may be a solution to attrition. The reminders sent to the user in real time may increase the completion of the homework, which has been shown to be a predictor of the outcome of the intervention [98, 99]. In addition, previous research using interventions administered over the Internet has found that adding professional support improves the adherence and increases the effect size of interventions [100].

The strengths of this clinical trial include the inclusion of a comparison group with random assignment, investigator-blinded assessments, clear presentation of inclusion and exclusion criteria, valid diagnostic and assessment methods, adequate sample size to offer statistical power, and a clear description of statistical methods. Therefore, it has all the characteristics of the most rigorous studies, which involve a randomized, prospective clinical trial, called type I, according to Nathan and Gorman criteria [101]. In addition, the completion of between-session homework promotes the transfer and generalization of trained skills. Finally, the trial will be conducted in the community context, so that its results will have a higher level of generalization to the general population.

Following the recommendations of the National Institute of Mental Health Psychosocial Intervention Development Workgroup [102] and the New Freedom Commission on Mental Health [103], this alternative format to traditional face-to-face programs to administer psychological interventions will increase the accessibility of psychological interventions and will increase the tools of professionals to reach a greater number of people. The advantages of interventions administered through video games include anonymity, savings due to reduced travel time, the ability of the participant to choose the moment of application (eliminating waiting time and appointments), lower stigma involved in playing a video game instead of attending a conventional therapy, and low cost [96].

Although there is currently a proliferation of commercial video games for the promotion of health and some studies have researched their effects [104], in many cases they have been designed for entertainment and very few have proven their efficacy scientifically [96]. This study may help reduce the dangers associated with the lack of quality control of video games currently available, providing an evidence-based intervention to promote active aging.

This study will provide information on the efficacy of an intervention for active aging and explore the use of alternative formats to increase the accessibility of therapies. It is an innovative study that will change the way in which treatments are received and reach a greater number of potential users, including those who are least likely to be served in traditional service centers and formats. The results of this study will benefit a large number of current older people and even more in the future, as the number of older adults with technological skills continues to increase. Considering the high social and economic costs of depression and cognitive impairment [3, 4], the implications for public health are significant.

## Trial status

Recruitment start: December 28, 2020.

Study completion: September 30, 2023.

## Supplementary information

**Additional file 1.** SPIRIT 2013 Checklist: Recommended items to address in a clinical trial protocol and related documents.

## Data Availability

Researchers will report study results through publications. The data supporting these findings will be presented in the main publications, and the datasets used during the study can be obtained from the corresponding author on reasonable request.

## Abbreviations

ATQ: Automatic Thoughts Questionnaire
BMI: Body mass index
BPAAT: Brief Physical Activity Assessment Tool
CBI-V: Cognitive-behavioral intervention administered through an online interactive multimedia video game with smartphone-based app companion
CES-D: Center for Epidemiologic Studies Depression Scale
CG: Control group
CSQ-8: Client Satisfaction Questionnaire
DMC: Data Monitoring Committee
Duke-UNC-11: Duke-UNC Functional Social Support Questionnaire
EG: Experimental group
EROS: Environmental Reward Observation
GHQ-12: General Health Questionnaire
GPPAQ: General Practice Physical Activity Questionnaire
M.I.N.I.: Mini International Neuropsychiatric Interview
MMQ: Multifactorial Memory Questionnaire
MMSE: Mini-Mental State Examination
REAP-S: Rapid Eating and Activity Assessment for Participants-Shortened Version
SF-36: 36-Item Short-Form Health Survey
SHI: Sleep Hygiene Index
